# Supplementary material for: Comparison of revised EWGSOP2 criteria of sarcopenia in patients with cancer using different parameters of muscle mass
Source: PLoS One. 2021 Sep 14;16(9):e0257446. doi: 10.1371/journal.pone.0257446 (PMC8439478; doi:10.1371/journal.pone.0257446)
Supplement: Attachment — Submitted filename: responsetoreviewers.docx [file pone.0257446.s001.docx]

Ana Paula Trussardi Fayh

Departament of Nutrition, Federal University of Rio Grande do Norte

Campus Universitário BR 101, Natal 59072-790 Brazil

Phone/Fax: +55 84 3342-2291

E-mail: [apfayh@yahoo.com.br](mailto:apfayh@yahoo.com.br)

February 17^th^, 2021.

**RESPONSE LETTER**

Manuscript PONE-D-20-33755

Comparison of revised EWGSOP2 criteria of sarcopenia in patients with cancer using different parameters of muscle mass

Dear Stephen E Alway, Ph.D.
Academic Editor:

Thank you for your email enclosing the reviewers’ comments. We have carefully reviewed the comments and have revised the manuscript accordingly. Our responses are given point-by-point below. Changes to the manuscript are tracked.

Sincerely,

Ana Paula Trussardi Fayh, PhD

Editor comments:

1. Please ensure that your manuscript meets PLOS ONE's style requirements, including those for file naming. The PLOS ONE style templates can be found at

<https://journals.plos.org/plosone/s/file?id=wjVg/PLOSOne_formatting_sample_main_body.pdf>

<https://journals.plos.org/plosone/s/file?id=ba62/PLOSOne_formatting_sample_title_authors_affiliations.pdf>

**Response:** The manuscript meets the style and templates provided by the journal.

2. Thank you for stating the following in the Acknowledgments Section of your manuscript:

"This study was financed in part by the Coordenação de Aperfeiçoamento de
306 Pessoal de Nível Superior - Brasil (CAPES) - Finance Code 001."

We note that you have provided funding information that is not currently declared in your Funding Statement. However, funding information should not appear in the Acknowledgments section or other areas of your manuscript. We will only publish funding information present in the Funding Statement section of the online submission form.

Please remove any funding-related text from the manuscript and let us know how you would like to update your Funding Statement. Currently, your Funding Statement reads as follows:

"The author(s) received no specific funding for this work."

 Please include your amended statements within your cover letter; we will change the online submission form on your behalf.

**Response:** Thank you for the comment. The statement was included in the submission form and removed from the manuscript. The cover letter was also changed accordingly.

Reviewers' comments:

**Reviewer #1**:

‘Comparison of revised EWGSOP2 criteria of sarcopenia in patients with cancer using different parameters of muscle mass’ is a research article that is a pioneer study evaluating the accuracy of sarcopenia-based CC, compared to CT images.
This study is very valuable because it highlights the fact that calf circumference measurement can be used to muscle mass assessment for sarcopenia diagnosis in cancer patients, instead of computed tomography (CT). Calf circumference measurement is a simple, easy and cheap procedure, in opposite to CT, which is costly method and requiring specialized staff to operate it. The paper reads well.

I would only recommend to widen the description of the use of the SARC-CalF questionnaire in research on sarcopenia around the world, because interest in this tool has grown significantly in recent years (lines 256-258).

Please refer to the following articles:

1) Yang M, Hu X, Xie L, et al. Screening sarcopenia in community-dwelling older adults: SARC-F vs SARC-F combined with calf circumference (SARC-CalF). J Am Med Dir Assoc. 2018;19(3):277.e1-277.e8. doi:10.1016/j.jamda.2017.12.016

2) Krzymińska-Siemaszko R, Deskur-Śmielecka E, Kaluźniak-Szymanowska A, Lewandowicz M, Wieczorowska-Tobis K. Comparison of Diagnostic Performance of SARC-F and Its Two Modified Versions (SARC-CalF and SARC-F+EBM) in Community-Dwelling Older Adults from Poland. Clin Interv Aging. 2020; 15: 583–594.

3) Mo Y, Dong X, Wang XH. Screening accuracy of SARC-F combined with calf circumference for sarcopenia in older adults: a diagnostic meta-analysis. J Am Med Dir Assoc. 2020;21(2):288–289. doi:10.1016/j.jamda.2019.09.002

**Response:** Thank you for the recommendation. Additional informations were included in the manuscript to refer to these studies.

**Reviewer #2**: Reviewer #2: This manuscript reports the comparison of sarcopenia in cancer patients using computed tomography and calf circumference. Overall the study is important and it will certainly add to the current evidence on the potential of using a simpler method to measure muscle mass in cancer patients. However, I have some minor comments on the manuscript, which are outlined below,

Abstract
• Please write L3 in full when it's first mentioned in the text, i.e. "third lumbar vertebra".

**Response:** Thank you for observation.
• Mean ± “standard deviation” age of 70.6 ± 7.4 years?

**Response:** Thank you for your observation, it was corrected according to the suggestion.


Materials and Methods
• Was this trial registered in clinical trials registration? If so, please include this information in the Methods section.

**Response:** Thank you for your observation. Considering the fact that the design of the study is cross-sectional (observational), no clinical trial registration was performed.


Design and subjects
• Lines 83-84: Please include the institution of the ethics board.

**Response:** Thank you for your observation. The institution was included according to the suggestion.

Anthropometric evaluation
• Lines 96-99: How many measurements were taken for the calf circumference, e.g. once or twice?

**Response:** Thank you for your observation. The information about the measurements was corrected and included in the manuscript (not in the dominant leg; both leg calf muscle circumferences were measured).

Muscle strength assessment
• Lines 104-105: Is there any reason why the handgrip strength of the non-dominant hand was captured as well?

**Response:** Thank you for your valuable observation. There is a mistake in this information, because handgrip strength was measured in both arms.


Results
• Table 1: Please include the definition for each of the BMI categories.

**Response:** Thank you for your observation. The definition was included in the footnote Table.

• Line 161: Should this read "Chi-square test"?

**Response:** Thank you for your observation, the term was modified.

• Line 180: Please write the abbreviations in full when first mentioned.

**Response:** Thank you for your observation. The abbreviations were defined in following sentences: BMI (line 97), CC (line 68), SMA (line 119), SMI (line 121), SMD (line 127) and HGS (line 107)

• Table 2: It will be good to include the prevalence of low handgrip strength, low MM, and low CC in this table. Thank you.

Response: Thank you for the recommendation to include these prevalences. However, we decided to present this information in the text (line 181-182).

• Line 183: Please change “according” to "according to".

**Response:** Thank you for your observation, the term was changed.

• Table 2 header: Please include the denominator for all the "No" and "Yes" categories, e.g. Sarcopenia by low MM, Yes: "26 of 108 patients = 24.1%".

**Response:** Thank you for your observation, the number of subjects was added on the title of Table.

• Line 194: Should this read "moderate"?

**Response:** Thank you for your observation, we corrected the word in the sentence.

• Table 3: Are all the kappa p-values referring to "p<0.001"?

**Response:** Thank you for your observation. Yes, all kappa p-values are p < 0.001. We changed the manuscript accordingly.

Discussion
• Lines 250-251: Suggest using another term for 'patients at risk of death'.

**Response:** Thank you for your observation, we changed the term in the sentence limiting to the studied population.

• Line 256: Should this read 'researchers"?

**Response:** Thank you for your observation, we corrected the word in the sentence.
