# Supplementary material for: Comparison of revised EWGSOP2 criteria of sarcopenia in patients with cancer using different parameters of muscle mass
Source: PLoS One. 2021 Sep 14;16(9):e0257446. doi: 10.1371/journal.pone.0257446 (PMC8439478; doi:10.1371/journal.pone.0257446)
Supplement: Attachment — Submitted filename: responsetoreviewers.docx [file pone.0257446.s002.docx]

Ana Paula Trussardi Fayh

Departament of Nutrition, Federal University of Rio Grande do Norte

Campus Universitário BR 101, Natal 59072-790 Brazil

Phone/Fax: +55 84 3342-2291

E-mail: [apfayh@yahoo.com.br](mailto:apfayh@yahoo.com.br)

June 22^nd^, 2021.

**RESPONSE LETTER**

Manuscript PONE-D-20-33755R1

Comparison of revised EWGSOP2 criteria of sarcopenia in patients with cancer using different parameters of muscle mass

Dear Stephen E Alway, Ph.D.
Academic Editor:

Thank you for your email enclosing the reviewers’ comments. We have carefully reviewed the comments and have revised the manuscript accordingly. Our responses are given point-by-point below. Changes to the manuscript are tracked.

Sincerely,

Ana Paula Trussardi Fayh, PhD

Reviewers' comments:

**Reviewer #1**

Thank you for considering my comments. I recommend publishing this revised version of manuscript titled Comparison of revised EWGSOP2 criteria of sarcopenia in patients with cancer using different parameters of muscle mass.

**Response:** Thank you for your comments; we appreciate your contributions to our manuscript.

**Reviewer #3**

Double check all abbreviations are used consistently in paper. E.g. “Muscle mass” has frequently been abbreviated to MM. However, in some cases it still remains written in full.
**Response:** Thank you for your observation; we have double-checked the main document and now all mentions of muscle mass after the abbreviation are abbreviated in the manuscript.

Abstract:
- Please introduce European Work Group on Sarcopenia in Older People (EWGSOP) in abstract before referring to abbreviation.
**Response:** Thank you for point this out. We have included the meaning of the abbreviation in the first mention.

- Line with “prevalence of sarcopenia by the EWGSOP2” – suggest revising to “according to the EWGSOP2 criteria.”

**Response:** Thank you for your comment, we have adjusted the sentence accordingly.

- Line with “evaluate the accuracy of sarcopenia using low CC considering MM assessment by computed tomography (CT)…” – suggest revising to “evaluate the accuracy of sarcopenia using low CC relative to MM assessment…”
**Response:** Thank you for your observation. We have changed the main document.

- Line “Data included sociodemographic, clinical and anthropometric variables.” – suggest deleting from abstract if you need to reduce word count. I don’t think this is necessary.

**Response:** Thank you for your observation. However, there was no need to reduce the word count after the changes in the abstract, the word count remains according to the journal guidelines (not exceed 300 words).

- Suggest adding one additional statement at the end of the abstract or slightly revise the current last statement of the abstract. What is the key takeaway message from this study, i.e. what is the primary reason people will cite this paper? E.g. Measuring CC and HGS may serve as a useful alternative to identify sarcopenia in people with cancer relative to relying on CT imaging to diagnose low MM.

**Response:** Thank you for your suggestion. We included additional information in the conclusion of the abstract to support the takeaway message of our study.

Introduction:
-Line 60: remove capitalisation of sarcopenia.

**Response:** Thank you for your observation. The capitalization was removed.

Methods:
- Line 90: This needs further clarification. How were patients approached for the study? Was there a set time period for recruitment, e.g. mm-yy to mm-yy?
**Response:** Thank you for your comments. We have included this information in the manuscript (lines 94 to 96).

- Line 101: Was the calf measurement performed by the same researcher?
**Response:** Thank you for your observation. Three trained researchers performed the calf measurement. This information was added in the revised manuscript (line 102).

- Line 103: After three measures of CC were taken, was the average then calculated across both legs or was it the maximum value that was used? This needs to be clearly articulated to allow for study replication or translation into practice.
**Response:** Thank you for your comments. After the three measures, it was used the maximum value. The additional information was included in the manuscript (line 110).

Results:
- Line 152: Was there a primary reason patients were unable to have their calf assessed? A little further detail might be helpful here.

**Response:** Thank you for your observation. The patients were unable to have their calf assessed because of the presence of leg edema or amputation. Additional information is provided in the revised version of the manuscript (Methods section, line 89 and Results section, line 159).

- Line 160: I would not refer to this as a nutritional evaluation, as that would require a more comprehensive assessment beyond BMI alone. Suggest revising this to: According to BMI categories, 47.2% of patients were classified as having a normal body weight and 30.6% were considered overweight.”

**Response:** Thank you for your suggestion. We have agreed with your comment and changed the manuscript according to your recommendation.

- Line 202-207: This is a very long sentence. Suggest condensing or breaking into two or more statements for clarity. Please also double check use of brackets. One seems to be missing before “all p-value <0.001.”

**Response:** Thank you for your observation. We have revised the sentence in the manuscript.

Discussion:
- Line 228: This sentence is a bit difficult to follow. Try to be very clear here. E.g. “The main finding of the present study is that CC can be used to diagnose sarcopenia in elderly patients with cancer and has similar accuracy to assessing MM using CT imaging.”
**Response:** Thank you for your comment. However, the sentence in the document is “The main finding of the present study points out that CC can be used, with good accuracy, as a MM marker to diagnose sarcopenia in elderly patients with cancer.”

-Line 230: Remove “In fact”

**Response:** Thank you for your observation. We have removed the sentence in the revised manuscript.

- The utility of measuring CC versus relying on sophisticated and costly imaging techniques to identify low MM should be highlighted. One of the most important findings of this study is that a relatively quick, cost-effective and easy measurement can help identify sarcopenia. I suggest highlighting this more in the discussion.

**Response:** Thank you for your suggestion. We agreed and included a sentence in the discussion section to highlight the importance of the use of CC (lines 237 to 238).
